# Symptomatic Management of Fever in Children: A National Survey of Healthcare Professionals’ Practices in France

**DOI:** 10.1371/journal.pone.0143230

**Published:** 2015-11-23

**Authors:** Nathalie Bertille, Gerard Pons, Babak Khoshnood, Elisabeth Fournier-Charrière, Martin Chalumeau

**Affiliations:** 1 Inserm U1153, Obstetrical, Perinatal and Pediatric Epidemiology Research Team (Epopé), Center for Epidemiology and Statistics Sorbonne Paris Cité (CRESS), Paris Descartes University, Paris, France; 2 Sorbonne Universités, UPMC Univ Paris 06, Paris, France; 3 Department of General Pediatrics, Hôpital Necker-Enfants Malades, AP-HP, Paris, France; 4 Clinical Pharmacology, Groupe hospitalier Cochin-Broca-Hôtel Dieu, AP-HP Paris-Descartes University, Paris, France; 5 Inserm U663 Pediatric epilepsies and brain plasticity, Paris, France; 6 Pain center, Hôpital Bicêtre, AP-HP, Le Kremlin Bicêtre, France; Centre Hospitalier Universitaire Vaudois, FRANCE

## Abstract

Despite the production and dissemination of recommendations related to managing fever in children, this symptom saturates the practices of primary healthcare professionals (HPs). Data on parent practices related to fever are available, but data on HPs’ practices are limited. We studied HPs’ practices, determinants of practices and concordance with recommendations in France. We conducted a national cross-sectional observational study between 2007 and 2008 among French general practitioners, primary care pediatricians and pharmacists. HPs were asked to include 5 consecutive patients aged 1 month to 12 years with acute fever. HPs completed a questionnaire about their practices for the current fever episode. We used a multilevel logistic regression model to assess the joint effects of patient- and HP-level variables associated with this behavior. In all, 1,534 HPs (participation rate 13%) included 6,596 children (mean age 3.7 ± 2.7 years). Physicians measured the temperature of 40% of children. Primary HPs recommended drug treatment for 84% of children (including monotherapy for 92%) and physical treatment for 62% (including all recommended physical treatments for 7%). HPs gave written advice or a pamphlet for 13% of children. Significant practice variations were associated with characteristics of the child (age, fever level and diagnosis) and HP (profession and experience). In France, despite the production and dissemination of national recommendations for managing fever in children, primary HPs’ observed practices differed greatly from current recommendations, which suggests potential targets for continuing medical education.

## Introduction

Fever is usually related to a rapidly self-limiting viral infection and may be associated with important discomfort [1, 2]. However, this symptom represents a large part of the practice of healthcare professionals (HPs) in primary care, such as general practitioners (GPs), office-based pediatricians, and pharmacists, and clinics as well as pediatric emergency departments [2–4]. Misconceptions by parents about rare serious causes such as severe bacterial infections beginning with isolated fever and possible association with convulsions have resulted in a “phobia” about fever [1, 5, 6]. To rationalize the symptomatic management of fever in children, several national health agencies and medical societies have produced and disseminated clinical guidelines [2–4, 7–9] (Table 1). We have recently shown, as did others, that parents’ practices were in poor concordance with these recommendations, notably for physical and drug treatments [10–15]. Discordance between parents’ practices and recommendations was associated with some characteristics of parents (educational level, profession), children (age, temperature level), and HPs (profession, experience) [10]. To prepare educational interventions for improving the quality of care of children, we need to study HPs’ practices to understand the reasons for the gap between recommendations and practices.

**Table 1:** Healthcare professionals’ practices for fever in children and concordance with recommendations.

	AAP	NICE	Afssaps	CPS	General practitioners (n = 3,270)*	Pediatricians (n = 1,596)*	Pharmacists (n = 1,730)*	Total (n = 6,596)*
					**N(%)**	**N(%)**	**N(%)**	**N(%)**
**Drug treatment**					3,187(97.5%)	1,469(92.0%)	864(49.9%)	5,520(83.7%)
***Monotherapy*** ^c^		***X***	***X***	***X***	***3*,*006(94*.*3%)***	***1*,*313(89*.*4%)***	***781(90*.*4%)***	***5*,*100(92*.*4%)***
**Physical treatments**								
N^‘^ (%) of available data					3,110(95.1%)	1,438(90.1%)	1,507(87.1%)	6,055(91.8%)
**At least 1 physical treatment**					1,708(54.9%) ^μ^	1,061(73.8%)	994(66.0%)	3,763(62.2%)
**Treatments**								
Give a cool bath					329(10.6%) ^μ^	126(8.8%) ^μ^	341(22.6%) ^μ^	796(13.1%) ^μ^
Avoid overdressing ^a^	**X**		**X**	**X**	1,158(37.2%) ^μ^	837(58.2%) ^μ^	605(40.2%) ^μ^	2,600(42.9%) ^μ^
Oral hydration ^a^	**X**	**X**	**X**	**X**	1,112(35.8%) ^μ^	702(48.8%) ^μ^	555(36.8%) ^μ^	2,369(39.1%) ^μ^
Avoid an overheated atmosphere^a^	**X**	**X**	**X**		377(12.1%) ^μ^	235(16.3%) ^μ^	260(17.3%) ^μ^	872(14.4%) ^μ^
Other^b^					188(6.1%) ^μ^	125(8.7%) ^μ^	141(9.4%) ^μ^	454(7.5%) ^μ^
At least 1 of 3 recommended physical treatments					1,586(51.0%) ^μ^	997(69.3%) ^μ^	861(57.1%) ^μ^	3,444(56.9%) ^μ^
***Three recommended physical treatments*** ^c^	***X***		***X***		***181(5*.*8%)*** ^μ^	***145(10*.*1%)*** ^μ^	***78(5*.*2%)*** ^μ^	***404(6*.*7%)*** ^μ^
**Written advice or pamphlet**					332(10.2%)	370(23.2%)	132(7.6%)	834(12.6%)

*no. of children recruited.

AAP, American Academy of Pediatrics; NICE, UK National Institute for Health and Clinical Excellence; Afssaps, French Drug Agency; CPS, Canadian Paediatric Society

^μ^ Percentage calculated on available data (n’)

^a^ Recommended physical treatments in our study.

^b^ Increase heating, not aerate the room, use a ventilator, dress the child, use a wet sponge, humidify the room.

^c^ Concordance with recommendations of our study.

Observational studies comparing HPs’ practices for fever in children to guidelines have reported an overuse of drug treatment and combined pharmacological treatments (acetaminophen plus ibuprofen) [5, 16–19]. However, these studies did not provide evidence of current HPs’ practices because they pre-dated the publication and diffusion of new recommendations, which might have had some impact [5, 16, 20–23], or were potentially biased by single-center [20, 24] or hospital-based recruitment [16, 20, 21, 24]. Furthermore, many studies focused on a specific medical profession [16–18, 20, 22, 23, 25–28] or pharmacists [23, 28], but the question is relevant to all HPs involved in primary care, and many studies were based on clinical scenarios and not observed practices [17–19, 23, 26, 29].

To identify targets of educational interventions for improving the quality of care of children, we aimed to explore the practices of primary care HPs in managing fever in children by using a national, cross-sectional survey in France.

## Methods

### General methodology

The study was previously described in detail [10]. In summary, we performed an observational national study over 8 months from November 2007 to June 2008. We contacted 4,163 GPs with pediatric patients (detected by pediatric vaccine prescriptions) from the national commercial panel of HPs, ICOMED (a panel representing more than 50% of French physicians in 2005 aimed at studying physicians’ preferences for drugs and treatments [30]); a random sample of French primary care pediatricians; and 4,946 pharmacists working in the same geographical area as a responding physician. Responding physicians and pharmacists were asked to include 5 consecutive patients from 1 month to 12 years old with fever for up to 48 hr who were accompanied to the HP visit by a family member.

Consent from both parents and HPs was obtained before inclusion and documented by completion of a questionnaire. The study was approved by the local ethics committee (Comité de Protection des Personnes Ile-de-France III, N°AT128).

### Instrument and data collected

The instrument used was a questionnaire with both open and closed questions that was developed by 3 experts (EFC, GP, and MC, co-authors of this study) in the fields of general pediatrics, pain in children, pediatric pharmacology, and epidemiology and tested with a convenience sample of investigators. The questionnaire included a first part to be completed by accompanying parents just before the consultation with the physician or advice by the pharmacist and the second part completed at the end of the consultation or after advice was given by the HP, who had access to the part completed by the parent. Data collected for all patients included the parent relationship with the child (mother, father, both or other), parents’ professional and educational levels, parents’ practices for the current episode (temperature measurement method and physical treatment), child characteristics (age, gender, birth rank, and main symptoms, including maximal temperature), and HP characteristics (practice location [urban, semi-urban, rural] and experience) and practices (prescriptions and advice to manage fever). Physicians were also asked to give information about temperature measurement during the consultation and the final diagnosis (categorized as respiratory illness, teething, sore throat, isolated fever, gastroenteritis, rash, influenza, rhinopharyngitis, otitis or other). Given current practices in France, for patients recruited by pharmacists, no data were collected on temperature measurement during the contact with the HP or on the final medical diagnosis.

### Statistical analyses

First, we briefly described the characteristics of the studied population, which are reported in detail elsewhere [10]. Second, we described HP practices for 4 key steps of the symptomatic management of fever in children: temperature measurement, drug treatment, physical treatment, and other general advice on fever management. Third, we compared these practices between GPs, office-based pediatricians and, when possible (see below), pharmacists, by using univariate then multivariate analyses after adjustment for potential confounders; these analyses allowed us to identify independent determinants of practices. All multivariate analyses involved a hierarchical regression model that took into account the hierarchical structure of the data (i.e., non-independence of the variables for the 5 patients included by each HP) and allowed us to include characteristics of parents and patients at the individual level (level 1) and the HP at the HP level (level 2). Among the numerous variables available for adjustment in multivariate multilevel models, variables were selected on the basis of theoretical causal diagrams and/or previous knowledge. Analyses involving temperature measurement and level during the contact with the HP and final medical diagnosis were restricted to the sample recruited by physicians (see explanation above). HPs’ practices were considered to be in concordance with recommendation (Table 1) when HPs suggested a drug monotherapy and the use of all 3 physical treatments concomitantly (oral hydration and avoiding overdressing and an overheated atmosphere). Analyses involved use of Stata v11 (StataCorp, College Station, TX, USA).

## Results

### Population characteristics

The sample included 6,596 children recruited by 757 GPs (18%), 373 pediatricians (16%) and 404 pharmacists (8%), for a mean participation rate of HPs of 13%. HPs had 19 years’ experience, on average, and 60% practiced in an urban area (47% of GPs, 94% of pediatricians and 52% of pharmacists, P <0.001). The mean age of patients was 3.7 ± 2.7 years, and 55% were male. The main other symptoms associated with fever were pain (64%), flu-like symptoms (according to parents, 49%) and cough (46%).

### HPs’ practices

During the consultation, the participating physicians measured the temperature for 40% of patients (44% for GPs and 33% pediatricians; P<0.001). After adjustment for patient and HP characteristics (S1 Table), temperature measurement by the physician was associated with lack of temperature measurement before the consultation by parents (adjusted odds ratio [aOR] 3.95), an inadequate temperature measurement method used by parents (aOR 1.65), lack of recent administration of a drug by parents (aOR 2.16), recruitment by a GP (aOR 3.41), and increased professional experience of HPs (aOR_15-23 years_ 2.04, aOR_>25 years_ 3.25). Patient age was not significantly associated with temperature measurement by the physician.

An antipyretic or analgesic was prescribed or recommended by HPs for 84% of patients: 97% for GPs, 92% pediatricians, and 50% pharmacists (P<0.001). Physicians prescribed drugs for antipyretic and analgesic action for 61% of patients, an antipyretic action alone for 37% and an analgesic action alone for 2%. In case of prescription (or advice), the first-choice drug prescribed by HPs was acetaminophen for 88% of patients (91% for pediatricians, 88% GPs, and 84% pharmacists; P<0.001), followed by ibuprofen for 11% (15% for pharmacists, 10% GPs, and 9% pediatricians; P<0.001). The drug recommended by HPs was the same as that already given by parents for 79% of patients. It was an oral formulation for 84% (syrup/powder, 82%; tablet, 3%) and a suppository for 17%. HPs recommended an adequate number of doses per day (i.e., 3 to 6 doses) for 97% of children, with a mean of 3.7 ± 0.6 doses per day. Monotherapy was prescribed for 92% of patients: 94% for GPs, 90% pharmacists, and 89% pediatricians (P<0.001). Monotherapy was prescribed at 3 to 6 doses per day for 89% of patients: 92% for GPs, 87% pediatricians and 84% pharmacists (P<0.001). After adjustment for patient and HP characteristics (S2 Table), the lack of prescription of any drug treatment was significantly associated with lower temperature (aOR_38.5–39°C_ 2.45), isolated fever (aOR 0.27), recruitment in a rural area (aOR_rural_ 0.43), and recruitment by a pediatrician (aOR 0.19). After adjustment for patient and HP characteristics (S3 Table), the prescription of bitherapy was associated with the accompanying parent being a farmer (aOR 2.64); a diagnosis of pharyngitis (aOR 2.17), gastroenteritis (aOR 0.52), otitis (aOR 2.27), or influenza (aOR 2.36); child's temperature ≥ 39°C (aOR_≥39°C_ 3.97); and recruitment by a pediatrician (aOR 2.02). Patient age was not significantly associated with prescription of bitherapy.

Among children recruited by pharmacists (n = 1,730), 51% already had a prior prescription after consultation with a physician. Children with a prior prescription from a physician were less likely to receive advice from the pharmacist than children without a prior prescription (28% vs. 77%, p <0.001). Pharmacists’ advice included a change in active molecule for 42% of patients: 23% from acetaminophen to ibuprofen and 15% from ibuprofen to acetaminophen. For 58% of patients for whom pharmacists recommended the same active molecule, the pharmacist’s recommendations included a formulation change for 25%: 9% from oral to rectal formulation, 8% from one oral formulation (syrup, tablet, and powder for solution) to another oral formulation, and 8% from a rectal to oral formulation.

At least one physical treatment to reduce the temperature was suggested by HPs for 62% of patients: 74% for pediatricians, 66% pharmacists, and 55% GPs (P <0.001). Physical treatment was suggested more often for the youngest patients: 69% for age 1 to 11 months, 69% for 1 to 2.5 years, 60% for 2.5 to 5 years and 51% for 5 to 12 years. These physical treatments were oral hydration (39%) and to avoid overdressing (43%) and an overheated atmosphere (14%) (Table 1). Oral hydration was suggested more often for the youngest patients: 42% for age 1 to 11 months, 44% for 1 to 2.5 years, 37% for 2.5 to 5 years and 33% for 5 to 12 years. All 3 treatments were concomitantly suggested for 7% of patients. A non-recommended physical treatment, to give a cool bath, was suggested by 13% of HPs: 23% pharmacists, 11% GPs and 9% pediatricians (P <0.001). Among patients recruited by physicians and after adjustment (S4 Table), the prescription of at least one recommended physical treatment was associated with the accompanying parent not being the father (aOR_mother_ 1.35, aOR_both_ 2.26, aOR_other_ 2.85), a young patient (aOR_2.5-5years_ 0.60, aOR_5-12years_ 0.29), a diagnosis of gastroenteritis (aOR 1.93), the lack of rash (aOR 0.30), a high temperature (aOR_≥39°C_ 2.30), and recruitment by a pediatrician (aOR 3.33).

HPs declared they had given oral advice for the management of fever to 85% of parents (86% for GPs, 85% pharmacists and 82% pediatricians, P = 0.001) and a pamphlet for 5% (8% for pharmacists, 4% pediatricians, and 3% GPs, P<0.001). Physicians delivered advice included in their written prescription to 11% of parents (20% for pediatricians and 7% GPs, P<0.001). Pharmacists recommended consultation with a physician for 53% of patients: 35% for children who had already been examined by a physician versus 70% if not (P<0.001). After adjustment (S5 Table), the delivery of advice in a written document (pamphlet and/or prescription) was associated with recruitment by a pediatrician (aOR 5.64) and increased experience of the HP (aOR_≥25 years_ 2.07).

## Discussion

In this national non-hospital-based cross-sectional prospective study, we report wide differences between current recommendations and observed practices of a large number of HPs (GPs, pediatricians and pharmacists) for the symptomatic management of fever in nearly 6,600 children. Contrary to recommendations [3] and as noted previously [30, 31], temperature, a major vital sign, was recorded for only 40% of children during consultations with physicians. A least one drug was almost systematically (> 90%) prescribed by GPs and pediatricians and frequently advised by pharmacists for patients without a previous consultation (> 70%). The level of concordance with recommendations for other observed HP practices was satisfactory and/or higher than previously observed: monotherapy (>92% vs. 27% to 91% [16–19, 26]), adequate number of doses per day (97%), and a bath (13%). Monotherapy may help to avoid drug misuse, and the American Academy of Pediatrics recently suggested that prescribing bitherapy may contribute to “fever phobia” [2]. These improvements are encouraging and indicate that a change in HP behavior is possible over time.

The first-choice drug was acetaminophen (mean 88%; within the range of previous reports, -60% to 99% [5, 16, 18, 19, 21, 26, 28, 32]), with a preference for the oral route (i.e., soluble or syrup) [17–19]. We observed that 40% of drugs were prescribed for an antipyretic action alone. This frequency may appear high because fever is now well recognized as a natural and beneficial reaction and international recommendations for antipyretic prescriptions have shifted during the last decades from a goal of absolute fever control to easing the child’s discomfort [2, 4, 33]. However, because a child could have signs of discomfort without necessarily having pain, there may be some overlap between prescriptions targeting discomfort and those targeting normothermia.

The safety profile of the available physical treatments (oral hydration and to avoid overdressing and an overheated atmosphere) is better than for pharmacological treatments, but the former were less frequently suggested (mean62%; within the range of previous reports, 58% to 87% [5, 17–19, 21, 24, 26]). These physical treatments were almost never associated (7%). Interestingly, oral hydration was suggested for only 42% of children with fever aged 1 to 11 months old, despite a higher risk of dehydration in this age group.

HPs poorly relied on written prescriptions (11%) and pamphlets (5%) to educate parents. Recommendations are lacking on the need to use pamphlets to guide parents and the evidence supporting the impact of written materials on patient outcomes is conflicting.

An unexpected finding of our study dealt with the practices of pharmacists for patients who had already consulted a physician and thus most probably came to the pharmacy to obtain medication rather than receive advice. In this case, we observed a high rate of change in active molecule between ibuprofen and acetaminophen (about 40%) and formulation for a given molecule (25%). These changes may be supported by concerns on effectiveness, safety, acceptability and palatability or parents’ perception of failure of the previously prescribed drug, but they could also have led to confusion for parents who did not ask for that change. Moreover, the changes from acetaminophen to ibuprofen by pharmacists without knowledge of the final medical diagnosis may be hazardous given the increasing concerns about the safety profile of ibuprofen in certain clinical conditions such as varicella and dehydration [34, 35].

We were able to study various determinants of HPs’ practices related to parents, the child or the HPs themselves. As in other studies, the temperature was not systematically measured by physicians (40% vs. 25% to 54%) [30, 31] and less often when parents had taken the temperature before the consultation and when this measurement was adequate (i.e., when parents measured the temperature with an electronic thermometer by a rectal, oral, auricular or axillary route). This situation may reflect a certain degree of confidence in the information provided by parents and a lack of time during busy consultations. Some diagnoses were associated with different patterns of practices. For example, particularly painful (otitis, pharyngitis) and feverish diseases were associated with prescriptions for bitherapy, as compared with gastroenteritis, which is potentially at risk of complications with nonsteroidal anti-inflammatory drugs [36]. Interestingly, the practices of more specialized HPs (pediatricians) were not necessarily in concordance with recommendations (e.g., bitherapy), although the literature suggested that pediatricians are more likely to follow recommendations [37–39] than GPs in various clinical situations. The association between the prescription of bitherapy and parents’ profession being a farmer can be related to the already-reported difficulty in implementing recommendations in rural areas [40]. The use of bitherapy when the child’s fever was particularly high may be explained by the persistence of “fever phobia” [18, 41].

Our present and previously published findings show important discrepancies between HPs’ practices and current recommendations [18, 19]. The literature has reported several barriers or facilitating factors affecting their implementation of guidelines [40, 42, 43]. Some factors are related to the guidelines themselves: their scientific validity, clinical applicability, adaptability to specific situations, and user-friendliness. Some are related to HPs: lack of awareness of guidelines, clinical inertia, and lack of outcome expectancy. Several suggestions have been made to overcome these barriers: short messages and easily accessible supports including electronic assistance; multifaceted approaches including participative ones; financial incentives; audits; and feedback [44]. In the context of our study, few of these recommendations were followed during the derivation and implementation of the French guidelines for the management of fever in children [45].

Our study has some limitations. It was conducted in 2008, and an evolution of knowledge and practices since then is likely. It was based on only reported practices, not observed ones. As well, the context may have modified practices (Hawthorne effect); and HPs had access to the answers of parents when they completed their part of the questionnaire. We did not have some information that may have been interesting to explore, such as the threshold for starting an antipyretic drug treatment recommended by HPs. Other limitations related to the design of our study were the lack of information about the precise reasons for changing the active molecule by pharmacists and exploring why HPs did not fully follow the recommendations. The selection of HPs was biased, and we could not compare features of participating (13%) and non-participating (87%) HPs, or compare GPs included in the commercial panel to their peers, but this commercial panel included more than 50% of French physicians. The number of parents who declined participation and the number of sibling children who participated were unknown. We could not estimate the strength and direction of the potential selection bias related to these limits. However, given the limitations of the existing evidence, which relied on hospital-based and/or single-center studies and/or collected data on a clinical scenario and not observed practices, we believe our results provide more accurate information on HPs’ practices than was previously available.

## Conclusions

This study shows that “fever phobia” persists among HPs, with important discrepancies between observed practices and recommendations. HPs’ practices were more in concordance with recommendations than practices previously reported and those among parents, which indicates that continuing medical education is an important medium in educating parents. Continuing medical education messages to improve the symptomatic treatment of fever in children could focus on the target of management, alleviating discomfort rather than fever control, and preventing dehydration among the youngest patients.

## Supporting Information

S1 Dataset (XLS)

S1 Table Factors associated with temperature measurement by a physician. (DOC)

S2 Table Factors associated with the prescription of antipyretic drug by a physician. (DOC)

S3 Table Factors associated with drug treatments in bitherapy for managing fever in children recruited by a physician. (DOC)

S4 Table Factors associated with prescription of at least one of 3 recommended physical treatments for managing fever in children recruited by a physician. (DOC)

S5 Table Factors associated with providing advice for the management of fever in written form. (DOC)
