# Supplementary material for: Symptomatic Management of Fever in Children: A National Survey of Healthcare Professionals’ Practices in France
Source: PLoS One. 2015 Nov 23;10(11):e0143230. doi: 10.1371/journal.pone.0143230 (PMC4658127; doi:10.1371/journal.pone.0143230)
Supplement: S1 Table — (DOC) [file pone.0143230.s002.doc]

S1 Table: Factors associated with temperature measurement by a physician.

| **Factors** | **No. of children** | **Univariate analysis** | |  | **Multivariate multi-level analysis** | |
| --- | --- | --- | --- | --- | --- | --- |
|  |  | ***OR*** | ***95% CI*** |  | ***aOR*** | ***95% CI*** |
| **Child’s age** |  |  |  |  |  |  |
| 1–11 months | 1,111 | 1 |  |  | 1 |  |
| 1–2.5 years | 1,246 | 1.06 | 0.79-1.43 |  | 1.01 | 0.74-1.38 |
| 2.5–5 years | 1,111 | 0.89 | 0.65-1.23 |  | 0.79 | 0.56-1.11 |
| 5–12 years | 1,079 | 0.95 | 0.69-1.32 |  | 0.79 | 0.56-1.11 |
| **Recent measurement of temperature by parents** |  |  |  |  |  |  |
| Yes | 3,509 | 1 |  |  | 1 |  |
| No | 1,038 | 5.51 | 4.14-7.35 |  | 3.95 | 2.90-5.39 |
| **Adequate temperature measurement method of parents ^a^** | |  |  |  |  |  |
| Yes | 3,995 | 1 |  |  | 1 |  |
| No | 552 | 3.09 | 2.24-4.27 |  | 1.65 | 1.16-2.36 |
| **Drug administered by parents before consultation** |  |  |  |  |  |  |
| Yes | 3,605 | 1 |  |  | 1 |  |
| No | 942 | 3.11 | 2.36-4.09 |  | 2.16 | 1.61-2.89 |
| **HP profession** |  |  |  |  |  |  |
| Pediatrician | 1,455 | 1 |  |  | 1 |  |
| General practitioner | 3,092 | 3.06 | 1.89-4.96 |  | 3.41 | 2.04-5.70 |
| **HP experience** |  |  |  |  |  |  |
| 0-14 years in practice | 1,247 | 1 |  |  | 1 |  |
| 15-23 years in practice | 1,719 | 1.61 | 0.91-2.83 |  | 2.04 | 1.13-3.67 |
| 24-54 years in practice | 1,581 | 2.25 | 1.26-4.01 |  | 3.25 | 1.77-5.97 |

OR, odds ratio; aOR, adjusted OR; 95% CI, 95% confidence interval; HP, healthcare professional

^a^ Temperature measured with an electronic thermometer by rectal, oral, aural or axillary route.
