# Supplementary material for: Symptomatic Management of Fever in Children: A National Survey of Healthcare Professionals’ Practices in France
Source: PLoS One. 2015 Nov 23;10(11):e0143230. doi: 10.1371/journal.pone.0143230 (PMC4658127; doi:10.1371/journal.pone.0143230)
Supplement: S2 Table — (DOC) [file pone.0143230.s003.doc]

S2 Table: Factors associated with the prescription of antipyretic drug by a physician.

| **Factors** | **No. of children** | | **Univariate analysis** | |  | | **Multivariate multi-level analysis** | | | |
| --- | --- | --- | --- | --- | --- | --- | --- | --- | --- | --- |
|  |  |  | ***OR*** | ***95% CI*** |  | | ***aOR*** | | ***95% CI*** | |
| **Child’s age** |  | |  |  |  | |  | |  | |
| 1–11 months | 1,151 | | 1 |  |  | | 1 | |  | |
| 1–2.5 years | 1,283 | | 1.09 | 0.67-1.75 |  | | 1.00 | | 0.62-1.63 | |
| 2.5–5 years | 1,124 | | 1.54 | 0.90-2.62 |  | | 1.22 | | 0.70-2.13 | |
| 5–12 years | 1,117 | | 1.72 | 0.98-3.03 |  | | 1.22 | | 0.67-2.22 | |
| **Teething** |  | |  |  |  | |  | |  | |
| No | 4,581 | | 1 |  |  | | 1 | |  | |
| Yes | 94 | | 0.45 | 0.16-1.28 |  | | 0.44 | | 0.15-1.26 | |
| **Pharyngitis** | |  |  |  | |  | |  | |  |
| No | | 3,985 | 1 |  | |  | | 1 | |  |
| Yes | | 690 | 0.71 | 0.43-1.17 | |  | | 0.66 | | 0.39-1.13 |
| **Rhinopharyngitis** | |  |  |  | |  | |  | |  |
| No | | 2,856 | 1 |  | |  | | 1 | |  |
| Yes | 1,819 | | 1.54 | 1.04-2.28 | |  | | 1.31 | | 0.85-1.99 |
| **Isolated fever** |  | |  |  | |  | |  | |  |
| No | 4,597 | | 1 |  | |  | | 1 | |  |
| Yes | 78 | | 0.21 | 0.08-0.57 | |  | | 0.27 | | 0.10-0.74 |
| **Temperature** |  | |  |  | |  | |  | |  |
| ≤38°C | 248 | | 1 |  | |  | | 1 | |  |
| 38-38.5°C | 1,030 | | 1.63 | 0.73-3.64 | |  | | 1.84 | | 0.82-4.12 |
| 38.5-39°C | 1,574 | | 2.16 | 0.98-4.74 | |  | | 2.45 | | 1.12-5.38 |
| >39°C | 1,823 | | 1.22 | 0.57-2.63 | |  | | 1.67 | | 0.77-3.59 |
| **HP profession** |  | |  |  | |  | |  | |  |
| General practitioner | 3,173 | | 1 |  | |  | | 1 | |  |
| Pediatrician | 1,502 | | 0.23 | 0.14-0.39 | |  | | 0.19 | | 0.10-0.36 |
| **HP practice location** |  | |  |  | |  | |  | |  |
| Urban | 2,896 | | 1 |  | |  | | 1 | |  |
| Largely rural | 1,080 | | 1.42 | 0.75-2.68 | |  | | 0.60 | | 0.29-1.21 |
| Rural | 699 | | 1.23 | 0.58-2.59 | |  | | 0.43 | | 0.18-0.98 |
| **HP experience** |  | |  |  | |  | |  | |  |
| 0–14 years in practice | 1,279 | | 1 |  | |  | | 1 | |  |
| 15–23 years in practice | 1,760 | | 0.75 | 0.37-1.39 | |  | | 0.85 | | 0.44-1.64 |
| 24–54 years in practice | 1,636 | | 0.57 | 0.29-1.10 | |  | | 0.76 | | 0.39-1.48 |
