# Supplementary material for: Symptomatic Management of Fever in Children: A National Survey of Healthcare Professionals’ Practices in France
Source: PLoS One. 2015 Nov 23;10(11):e0143230. doi: 10.1371/journal.pone.0143230 (PMC4658127; doi:10.1371/journal.pone.0143230)
Supplement: S3 Table — (DOC) [file pone.0143230.s004.doc]

S3 Table: Factors associated with drug treatments in bitherapy for managing fever in children recruited by a physician

| **Factors** | | **No. of children** | | | **Univariate analysis** | | |  | | **Multivariate multi-level analysis** | | | | |
| --- | --- | --- | --- | --- | --- | --- | --- | --- | --- | --- | --- | --- | --- | --- |
|  |  |  |  |  | ***OR*** | ***95% CI*** | |  | | ***aOR*** | | | ***95% CI*** | |
| **Child’s age** |  | | | |  | |  |  | |  | |  | | |
| 1–11 months | 1,029 | | | 1 | | |  | |  | | 1 | | |  |
| 1–2.5 years | 1,162 | | | 0.94 | | | 0.63-1.41 |  | | 0.89 | | 0.58-1.36 | | |
| 2.5–5 years | 1,021 | | | 1.33 | | | 0.88-2.00 |  | | 1.34 | | | 0.87-2.08 | |
| 5–12 years | 1,014 | | | 0.70 | | | 0.45-1.10 |  | | 0.77 | | | 0.47-1.27 | |
| **Accompanying parent profession** | | |  | |  |  | |  | |  | | |  | |
| Executive | | | 1,045 | | 1 |  | |  | | 1 | | |  | |
| Farmer | | | 123 | | 1.78 | 0.81-3.90 | |  | | 2.64 | | | 1.14-6.10 | |
| Craftsman/storekeeper | | | 333 | | 0.65 | 0.35-1.21 | |  | | 0.78 | | | 0.41-1.49 | |
| Employee | | | 1,478 | | 1.01 | 0.70-1.44 | |  | | 1.20 | | | 0.82-1.75 | |
| Salaried worker | | | 487 | | 0.53 | 0.30-0.93 | |  | | 0.77 | | | 0.42-1.40 | |
| Retired person | | | 81 | | 0.30 | 0.07-1.21 | |  | | 0.46 | | | 0.11-1.96 | |
| Unemployed | | | 679 | | 0.60 | 0.37-0.98 | |  | | 0.77 | | | 0.46-1.28 | |
| **Pharyngitis** | | |  | |  |  | |  | |  | | |  | |
| No | | | 3,613 | | 1 |  | |  | | 1 | | |  | |
| Yes | | | 613 | | 1.75 | 1.21-2.54 | |  | | 2.17 | | | 1.42-3.30 | |
| **Gastroenteritis** | | |  | |  |  | |  | |  | | |  | |
| No | | | 3,817 | | 1 |  | |  | | 1 | | |  | |
| Yes | | | 409 | | 0.32 | 0.16-0.62 | |  | | 0.52 | | | 0.25-1.05 | |
| **Influenza** | | |  | |  |  | |  | |  | | |  | |
| No | | | 3,806 | | 1 |  | |  | | 1 | | |  | |
| Yes | | | 420 | | 2.21 | 1.45-3.39 | |  | | 2.36 | | | 1.46-3.81 | |
| **Otitis** | | |  | |  |  | |  | |  | | |  | |
| No | | | 3,526 | | 1 |  | |  | | 1 | | |  | |
| Yes | | | 700 | | 1.96 | 1.39-2.75 | |  | | 2.27 | | | 1.55-3.32 | |
| **Temperature** | | |  | |  |  | |  | |  | | |  | |
| ≤38°C | | | 231 | | 1 |  | |  | | 1 | | |  | |
| 38-38.5 °C | | | 978 | | 1.58 | 0.59-4.21 | |  | | 1.49 | | | 0.54-4.08 | |
| 38.5-39°C | | | 1,514 | | 2.35 | 0.91-6.06 | |  | | 2.03 | | | 0.77-5.40 | |
| >39°C | | | 1,503 | | 5.50 | 2.14-14.11 | |  | | 3.97 | | | 1.50-10.47 | |
| **HP profession** | | |  | |  |  | |  | |  | | |  | |
| General practitioner | | | 2,912 | | 1 |  | |  | | 1 | | |  | |
| Pediatrician | | | 1,314 | | 2.43 | 1.66-3.56 | |  | | 2.02 | | | 1.31-3.11 | |
