# Supplementary material for: Symptomatic Management of Fever in Children: A National Survey of Healthcare Professionals’ Practices in France
Source: PLoS One. 2015 Nov 23;10(11):e0143230. doi: 10.1371/journal.pone.0143230 (PMC4658127; doi:10.1371/journal.pone.0143230)
Supplement: S4 Table — (DOC) [file pone.0143230.s005.doc]

S4 Table: Factors associated with prescription of at least one of 3 recommended physical treatments for managing fever in children recruited by a physician

| **Factors** | **No. of children** | **Univariate analysis** | |  | **Multivariate multi-level analysis** | |
| --- | --- | --- | --- | --- | --- | --- |
|  |  | ***OR*** | ***95% CI*** |  | ***aOR*** | ***95% CI*** |
| **Accompanying parent** |  |  |  |  |  |  |
| Father | 725 | 1 |  |  | 1 |  |
| Mother | 3,221 | 1.39 | 1.08-1.80 |  | 1.35 | 1.02-1.79 |
| Both parents | 225 | 2.28 | 1.40-3.72 |  | 2.26 | 1.27-4.03 |
| Other | 168 | 2.39 | 1.39-4.11 |  | 2.85 | 1.55-5.22 |
| **Child’s age** |  |  |  |  |  |  |
| 1–11 months | 1,070 | 1 |  |  | 1 |  |
| 1–2.5 years | 1,200 | 1.02 | 0.77-1.34 |  | 0.98 | 0.72-1.35 |
| 2.5–5 years | 1,043 | 0.66 | 0.49-0.89 |  | 0.60 | 0.43-0.83 |
| 5–12 years | 1,026 | 0.33 | 0.25-0.45 |  | 0.29 | 0.20-0.40 |
| **Gastroenteritis** |  |  |  |  |  |  |
| No | 3,918 | 1 |  |  | 1 |  |
| Yes | 421 | 1.43 | 1.03-1.97 |  | 1.93 | 1.32-2.82 |
| **Rash** |  |  |  |  |  |  |
| No | 4,268 | 1 |  |  | 1 |  |
| Yes | 71 | 0.42 | 0.20-0.92 |  | 0.30 | 0.13-0.73 |
| **Child’s temperature** |  |  |  |  |  |  |
| <38.5 °C | 1,250 | 1 |  |  | 1 |  |
| 38.5-39°C | 1,554 | 1.11 | 0.88-1.41 |  | 1.20 | 0.92-1.56 |
| >39°C | 1,535 | 1.84 | 1.43-2.36 |  | 2.30 | 1.72-3.06 |
| **HP profession** |  |  |  |  |  |  |
| General practitioner | 2,948 | 1 |  |  | 1 |  |
| Pediatrician | 1,391 | 3.11 | 2.08-4.64 |  | 3.33 | 2.06-5.37 |
