# Supplementary material for: Symptomatic Management of Fever in Children: A National Survey of Healthcare Professionals’ Practices in France
Source: PLoS One. 2015 Nov 23;10(11):e0143230. doi: 10.1371/journal.pone.0143230 (PMC4658127; doi:10.1371/journal.pone.0143230)
Supplement: S5 Table — (DOC) [file pone.0143230.s006.doc]

S5 Table: Factors associated with providing advice for the management of fever in written form

| **Factors** | **No. of children** | **Univariate analysis** | |  | **Multivariate multi-level analysis** | |
| --- | --- | --- | --- | --- | --- | --- |
|  |  | ***OR*** | ***95% CI*** |  | ***aOR*** | ***95% CI*** |
| **Accompanying parent** |  |  |  |  |  |  |
| Mother | 4,159 | 1 |  |  | 1 |  |
| Father | 948 | 0.68 | 0.48-0.96 |  | 0.73 | 0.51-1.05 |
| Both parents | 228 | 1.93 | 1.15-3.21 |  | 1.73 | 1.01-2.97 |
| Other | 242 | 1.02 | 0.58-1.79 |  | 1.00 | 0.53-1.89 |
| **Child’s age** |  |  |  |  |  |  |
| 1–11 months | 1,319 | 1 |  |  | 1 |  |
| 1–2.5 years | 1,484 | 1.05 | 0.75-1.47 |  | 1.13 | 0.78-1.62 |
| 2.5–5 years | 1,323 | 0.77 | 0.53-1.10 |  | 0.94 | 0.63-1.40 |
| 5–12 years | 1,451 | 0.53 | 0.36-0.79 |  | 0.68 | 0.45-1.04 |
| **No. of children in family** |  |  |  |  |  |  |
| 1 | 1,752 | 1 |  |  | 1 |  |
| 2 | 2,531 | 0.81 | 0.61-1.07 |  | 1.03 | 0.76-1.39 |
| ≥ 3 | 1,294 | 0.92 | 0.66-1.28 |  | 1.29 | 0.90-1.85 |
| **Temperature** |  |  |  |  |  |  |
| <38.5 °C | 1,701 | 1 |  |  | 1 |  |
| 38.5-39°C | 1,967 | 0.79 | 0.58-1.08 |  | 0.79 | 0.57-1.09 |
| >39°C | 1,909 | 1.24 | 0.91-1.70 |  | 1.14 | 0.82-1.58 |
| **HP profession** |  |  |  |  |  |  |
| General practitioner | 2,774 | 1 |  |  | 1 |  |
| Pediatrician | 1,363 | 6.69 | 3.99-11.21 |  | 5.63 | 3.03-10.48 |
| Pharmacist | 1,440 | 0.64 | 0.36-1.13 |  | 0.73 | 0.40-1.33 |
| **HP practice location** |  |  |  |  |  |  |
| Urban | 3,309 |  |  |  | 1 |  |
| Largely rural | 1,429 | 0.54 | 0.32-0.92 |  | 1.07 | 0.59-1.96 |
| Rural | 839 | 0.46 | 0.23-0.91 |  | 1.01 | 0.48-2.13 |
| **HP experience** |  |  |  |  |  |  |
| 0–14 years in practice | 1,705 | 1 |  |  | 1 |  |
| 15–23 years in practice | 1,951 | 1.34 | 0.75-2.39 |  | 1.10 | 0.60-2.01 |
| 24–54 years in practice | 1,921 | 2.83 | 1.61-4.99 |  | 2.07 | 1.15-3.76 |
